# A Land Use/Land Cover Based Green Development Study for Different Functional Regions in the Jiangsu Province, China

**DOI:** 10.3390/ijerph16071277

**Published:** 2019-04-10

**Authors:** Xiaomin Guo, Xiaowei Chuai, Xianjin Huang

**Affiliations:** School of Geography and Ocean Science, Nanjing University, Nanjing 210023, China; guoxiaomin0611@163.com (X.G.); hxj369@nju.edu.cn (X.H.)

**Keywords:** land use/land cover change, green development, carbon storage, functional region, low carbon land use

## Abstract

Land use/land cover (LULC) change can strongly affect carbon storage in terrestrial ecosystems. The rapid development of China’s economy has formed different functional regions. These functional regions profoundly affect land use patterns. Thus, assessing the carbon storage induced by LULC changes is significant for green development. Selecting the typical region of the Jiangsu Province as the study area, this study first examines the research associated with the regional functional characteristics and various high accuracy data and methods have been used to greatly improve the research accuracy. The results showed that from 1995 to 2015, approximately 10.26% of the entire land area had LULC type changes. Additionally, decreases in the built-up land expansion and ecological land were the main LULC change characteristics, which are mainly affected by socioeconomic development. The total carbon storage of the Jiangsu Province decreased by 714.03 × 10^4^ t and the four regions all presented decreasing carbon storage levels. The economically developed regions presented a more obvious loss of carbon. The region with small LULC changes had a lower carbon loss. The land transfer of cultivated land to built-up land is the main transfer type causing the carbon storage loss. This study investigates the human-environmental interactions from the new perspective of functional zoning and, thus, it enriches the comparative analysis of carbon storage in functional regions and provides references for the green development of a developing country’s developed areas.

## 1. Introduction

Carbon storage and its change in terrestrial ecosystems play an important role in the carbon cycle and are the focus of global climate change [1,2]. Lal (2008) reported that the total global carbon storage in terrestrial ecosystems can reach 2110 Pg [3], which is almost three times the amount of CO_2_ in atmospheric CO_2_. From a low-carbon development background, determining how to undertake the coordinated development and allocation of carbon emission tasks reasonably among the functional regions is particularly important for low-carbon development overall. Land use/land cover (LULC) change caused by human activities is the main factor influencing carbon storage in terrestrial ecosystems. Through altering vegetation cover and biomass, LULC changes can directly influence the carbon storage of vegetation. LULC changes can also affect the soil organic carbon (SOC) by altering the organic matter that returns to the soil [4,5], changing the regional hydrothermal condition and even local microclimate; although this process takes considerably longer compared to processes as vegetation carbon storage changes [6]. For example, grassland degradation and conversion to cultivated land always reduces the vegetation biomass and can increase the release of carbon into the soil [7,8], whereas changes from other land use types to woodland always increase carbon storage [9,10]. It has been recognized that LULC changes can significantly affect carbon storage because of the different capacities of accumulated carbon in the differing land use types [11]. 

Numerous studies considering carbon storage related to LULC changes have been published. Those studies mainly determined the carbon sequestration capacity for each LULC type and calculated the carbon storage changes caused by land use conversion [12,13,14,15,16]. In China, studies have been conducted on the national [17,18,19,20,21], provincial [22,23,24,25] and local scales [26,27,28]. However, few studies have focused on comparative studies of functional regions. In fact, functional zoning offers an alternative perspective to investigate the multiple means of human-environmental interactions and comparative studies of functional regions can be very meaningful. In addition, most of the studies primarily focused on single aspects such as vegetation carbon [29,30], SOC [31,32,33], or single ecosystems such as those of forests [34,35], cultivated land [36,37], etc. A comprehensive examination of both vegetation and SOC of all ecosystems is relatively rare. Additionally, previous studies have mostly been based on old carbon data or empirical data, especially the SOC data, which is based on the second national soil survey of the 1980s [38], which may not reflect the physical truth. Thus, new survey data are needed to examine the current SOC level. In addition, previous studies always used 1-km or 100-m grid land use maps [39,40], resulting in great bias. Hence, high-resolution grid maps need to be updated both in a timely and spatial way to reflect the current changing character and the corresponding induced carbon storage changes accurately.

This study can fill the research gap mentioned above, taking Jiangsu province as the study area, one of the most developed provinces of China. This study firstly undertakes a comparative research study associated with functional characteristics. The 30-m high-resolution land use type, new soil sampling map and other various data and methods have been used to improve the research accuracy. Our study will enrich the comparative analysis of carbon storage in the functional regions and provide meaningful references for the coordinated low-carbon development in the Jiangsu Province. The main objectives of this study were (1) to calculate the carbon density of each LULC type, (2) to examine the temporal-spatial changes in land transition and its driving forces and their effects on carbon storage, (3) to make a comparative analysis of carbon storage change related to LULC changes among functional regions, and (4) to provide appropriate references for the green development in the four functional regions.

## 2. Materials and Methods 

### 2.1. Study Area

**Figure 1:**
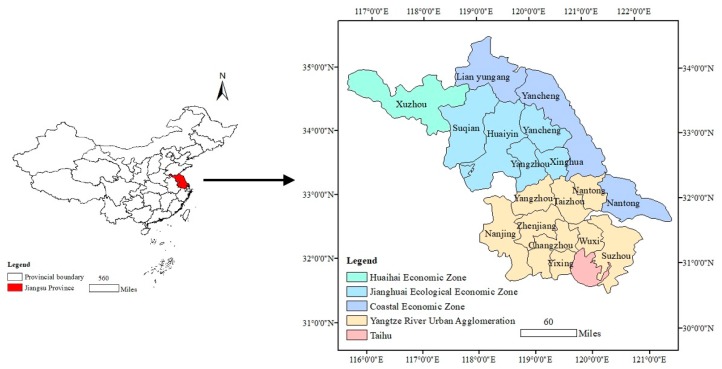
Location of the study area.

Jiangsu Province is located in eastern China; facing the Yellow River (Figure 1). This province lies between longitude 116°18′–121°57′, latitude 30°45′–35°20′ and has an area of 10.72 × 10^4^ km^2^. The topography is dominated by plains, it has the lowest terrain in China, and most of the areas are below 50 m. In recent years, the accelerating process of industrialization and urbanization has caused obvious land use changes, especially in the built-up land expansion, and these obvious LULC changes surely will drive regional carbon storage change.

Jiangsu Province is a major economic and cultural centre of China. The spatial disparity of Jiangsu Province has long been considered an economic gradient descending from the south to the north. In 2017, in order to promote the overall coordinated development, Jiangsu province promulgated a new key functional region strategy; the whole province was divided into four functional regions according to the regional characteristics, resource endowment, functional orientation, and by ignoring the geographical boundaries: Yangtze River City Group, the Coastal Economic Belt, the Huaihai Economic Region and the Jianghuai Ecological Economic Region. The Yangtze River City Group, consisting of economically developed cities with a good location and economic development advantages, is the engine for the economic development of the entire province; the Coastal Economic Belt, involving three coastal cities, is a potential growth pole; the Huaihai Economic Region regards Xuzhou as the centre to expand the depth of development in the Jiangsu Province; and the Jianghuai Ecological Economic Region, with the best ecological background and relatively backward economic development, is committed to building the ecological garden of Jiangsu, which mainly yields ecological advantages. Carbon storage related to land use change in the four functional regions varies obviously. 

### 2.2. Datasets and Pre-Processing

**Figure 2:**
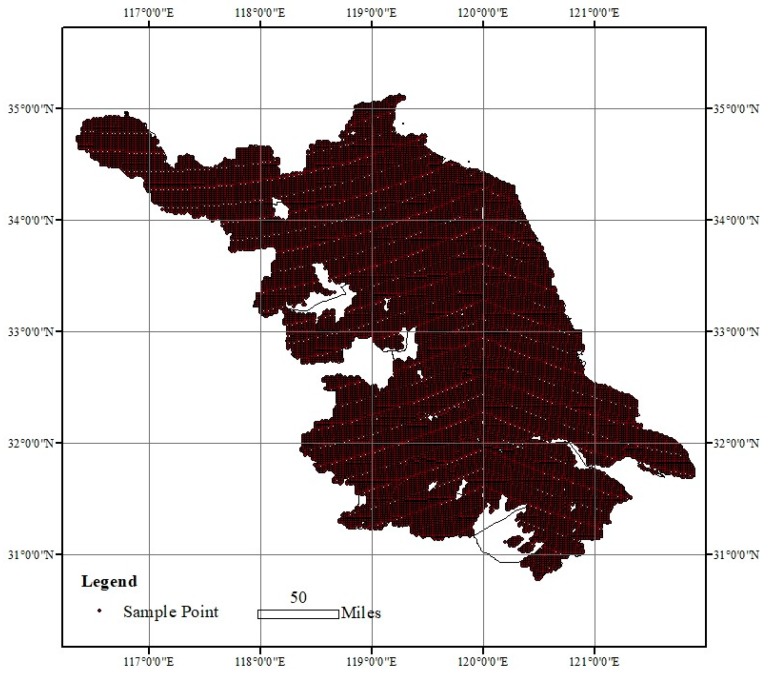
The distribution of soil sampling sites.

The following datasets and processing procedures were carried out in this study: (1) Land-use grids of 30 m × 30 m with periods of 2000, 2005, 2010 and 2015 were provided by the Data Center for Resources and Environmental Sciences, Chinese Academy of Sciences (RESDC) (http://www.resdc.cn). The data quality was improved and the comprehensive valuation accuracy of the first level of land use is >93% and that of the second level is >90% [41]. We used the first land use classification to analyze the land use changes of cultivated land, woodland, grassland, water areas, built-up land and unused land. The population change and economic development were known as the main drivers of land use changes [42], which were obtained from the “Jiangsu Statistical Yearbook”. (2) The soil sample data were obtained from the Jiangsu Geological Survey Institute and comprised of 24,186 samples of data of 0–20 cm soil profiles, with sampling times from 2001 and 2004, which were evenly distributed in the study area (Figure 2). Each soil sample has a record of latitude and longitude, soil organic carbon content (%), soil type, and bulk density. Detailed SOC testing and disposal processes can be obtained from the study of Liao et al. (2009) [43], including data on the surface soil layer (0–20 cm). (3) The vegetation-type map was compiled using data from the 2000s and was able to effectively describe the most recent vegetation distribution in the study area. (4) The forest data were from the fifth national forest inventory information, including vegetation type, vegetation area, forest age group and stock volume, and statistics on the forest area in the Jiangsu Province in 2005. (5) The crop economic yield data was acquired from the “2016 Jiangsu Province statistical yearbook”. (6) Some empirical data. The constant values a and b (Equation 1) were derived from the empirical data published in a previous study of Xu (2007) [44], who established linear regression equations between forest biomass and its volume based on 2304 forest sample plots in China. The economic coefficient and carbon conversion coefficient for each crop were from empirical data published in a paper by Li (2002) [45], who estimated the carbon storage in farmland ecosystems in China. Biomass data of various vegetation types were quoted from the average results of the research by Zong et al. [46], who studied the biomass of saltwater vegetation and sand vegetation on the coast of the Jiangsu Province.

### 2.3. Methods

#### 2.3.1. Vegetation Carbon Densities for Different LULC Types

##### Woodland

(1) $$C=\frac{\sum C_{ij}\times S_{ij}}{\sum S_{ij}},\;C_{ij}=B_{ij}\times 0.5,\;\mathrm{and}\;B_{ij}=a+bV_{ij}$$

where C represents the average vegetation carbon density of woodland, and $C_{ij}$ and $S_{ij}$ represent the vegetation carbon density and woodland area, respectively. $B_{ij}$ and $V_{ij}$ are the vegetation biomass density and volume density of tree-type *i* and tree-age *j*. a and b are constants according to the study by Xu et al. [44].

##### Cultivated Land

First, we calculated the biological yield of each crop; the formula is shown below:

(2) $$D_{W}=Y_{W}/H_{i}$$

where $Y_{W}$ represents the economic yield, and $D_{W}$ and $H_{i}$ are the biological yield and the economic coefficient, respectively.

Then, the carbon storage of each crop during the growth period was calculated (Table 1); the formula is as follows:

(3) $$C_{d}=C_{f}D_{w}=C_{f}Y_{w}/H_{i}$$

where $C_{d}$ represents the carbon storage of each crop, and $C_{f}$ represents the carbon conversion coefficient.

##### Other Lands

For grassland, we also used the biomass method to estimate the carbon storage, as grassland is mainly located along the coastline. Biomass data for various types of vegetation were quoted from the average results of the research by Zong et al. [46], who studied the biomass of saltwater vegetation and sand vegetation on the coast of the Jiangsu Province. According to the vegetation type, we can calculate the area for every vegetation type and we can then calculate the average vegetation carbon density of grassland. For water area and unused land, because their vegetation coverages are almost zero, we define their vegetation carbon densities to be zero. For built-up land, the main vegetation types are woodland and grassland. Therefore, we calculated the average vegetation carbon density of woodland and grassland and then multiplied it by the vegetation coverage rate of the Jiangsu Province as the vegetation carbon density of built-up land.

#### 2.3.2. Soil Organic Carbon Densities for Different LULC Types

SOC in the surface soil layer is more sensitive to LULC changes and this study only considered the soil surface at the 0–20 cm depth. To obtain the SOC densities for different LULC types, we first produced a soil sample distribution map according to the latitude and longitude of each soil sample and we then generated the soil organic carbon density (SOCD) distribution map covering the entire study area using the Kriging Interpolation Method of ArcGIS 10.3 (ESRI Inc., Redlands, CA, USA). Finally, we overlaid the SOCD distribution map and the LULC type map for 2015 and then conducted a statistical analysis to obtain the average SOCD for each LULC type using the Zonal Statistics as the table of ArcGIS 10.3.

#### 2.3.3. LULC Changes and Driving Forces Analysis 

A quantitative analysis is performed for the land use types: cultivated land $Y_{1}$, transportation land $Y_{2}$, and residential and industrial land $Y_{3}$. Driving forces are selected as below: gross domestic product (GDP) $X_{1}$, total population $X_{2}$, urbanization rate $X_{3}$, urban population $X_{4}$, fixed-asset investment $X_{5}$, urban per capita housing area $X_{6}$, rural per capita housing area $X_{7}$, rural population $X_{8}$, annual cargo capacity $X_{9}$, and annual passenger capacity $X_{10}$. Using the SPSS software, correlation analyses were used to analyze the relationships between land use type changes and the driving forces. 

#### 2.3.4. Carbon Storage Changes Caused by LULC Changes 

Based on the LULC images of Jiangsu Province in 1995 and 2015, we made a land transformation matrix from 1995 to 2015 by ArcGIS 10.3. Then we determined the carbon storage change based on the land transformation matrix and the SOCD and vegetation carbon density. The formula is as follows:

(4) $$T=\sum S_{ij}\times \Delta d_{ij}$$

where T is total carbon storage change; $S_{ij}$ is the area transferred from LULC type *i* to LULC type *j*; $\Delta d_{ij}$ is the carbon density change when LULC type *i* is transferred to LULC type *j*; *i* = 1,2,3,4,5,6, and *j* = 1,2,3,4,5,6 for the six LULC types including cultivated land, woodland, grassland, water area, built-up land and unused land.

## 3. Results

### 3.1. Carbon Densities of Different LULC Types 

Apart from SOC, the basic data for the vegetation carbon density calculation were for the entirety of Jiangsu Province as a unit; thus, the statistics on vegetation carbon density permitted no regional comparison (Table 2) and the vegetation carbon densities varied from 0.00 to 1.94 kg/m^2^ among the different LULC types. Woodland presented the highest value, with cropland following at 1.13 kg/m^2^, while the density dropped dramatically for the other LULC types, with grassland at 0.21 kg/m^2^, built-up land at 0.11 kg/m^2^, and 0.00 kg/m^2^ for both water area and unused land. 

Table 3 shows that SOCD differed among the LULC types, but the range of variation is much narrower than for the vegetation carbon densities. Although woodland has the highest biomass level, it did not always present the highest SOCD level; built-up land and water area can also present high SOCD levels. SOCD differences were also obvious among different functional regions. In terms of the average value, the average SOCD in the Yangtze River City Group had the highest value of 3.59 kg/m^2^ and the lowest value was in the Coastal Economic Belt (2.27 kg/m^2^). SOCD for the same LULC type also shows obvious regional differences; for example, the SOCD of grassland is much lower in the Coastal Economic Belt compared with those for other LULC types and other regions. 

**Figure 3:**
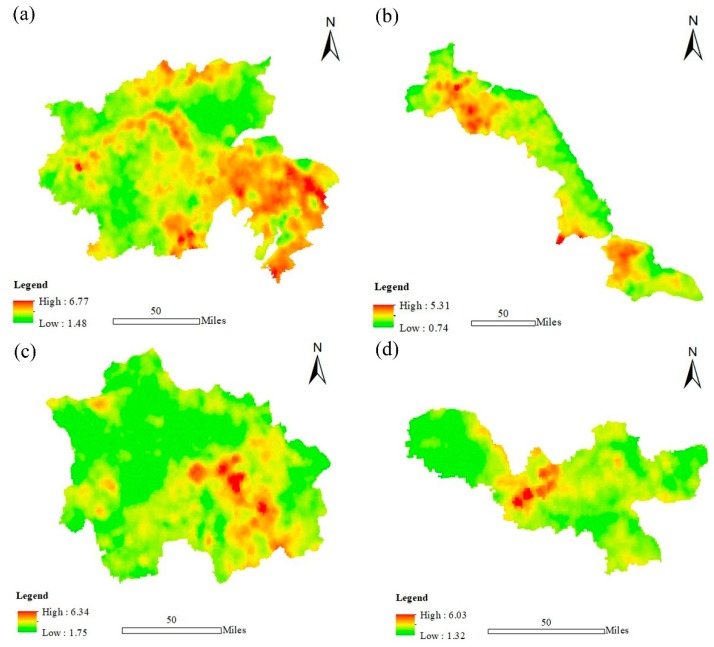
The spatial distributions of soil organic carbon density (SOCD) in four functional regions (kg/m^2^); (**a**), (**b**), (**c**) and (**d**) represent the Yangtze River City Group, Coastal Economic Belt, Jianghuai Ecological Economic Region and Huaihai Economic Region, respectively.

Figure 3 shows the SOCD distributions for the four functional regions. In the figure, we can find that the areas of high SOCD were mainly located in the Yangtze River City Group, whereas the values were relatively low for the other three regions. Individually, areas with relatively higher SOCD values in the Yangtze River City Group were mainly distributed in the southeast of this region. The SOCD values distributed in the centre and southeast of the Jianghuai Ecological Economic Region were relatively higher. The higher values of SOCD in the Huaihai Economic Region were mainly distributed in the centre of that region, and the other areas’ values were generally lower. In the Coastal Economic Belt, the relatively higher SOCD values were mainly distributed in the northwest and southeast of the region.

### 3.2. Quantitative Changes in LULC in the Four Functional Regions

Table 4 shows that the amounts of changes in LULC between 2005 and 2010 were more obvious. The built-up land expansion was obvious in the four functional regions, especially during the period of 2005 to 2010. Cultivated land area decreased between 1995 and 2015, contrary to the built-up land. Woodland and grassland both presented a decreasing trend overall between 1995 and 2015. The water area increased and decreased, although it increased overall. The built-up land area decreased most during the period of 2005 to 2010 in the Yangtze River City Group (2358.55 km^2^), accounting for 40.78% of the total built-up land area in 2005. The water area in both the Coastal Economic Belt and Jianghui Ecological Economic Region increased obviously, especially during the period of 2005 to 2010. The woodland area in the Huaihai Economic Region decreased between 1995 and 2015, with the greatest change rate of −22.62% during the period of 2005 to 2010.

### 3.3. Spatiotemporal Changes in LULC in the Four Functional Regions

Using ArcGIS 10.3, the land transfer between 1995 and 2015 was analyzed both in quantity and over space. Table 5 shows that a large amount of cultivated land was transferred to built-up land, which was the main LULC transfer type for all the functional regions. It was particularly obvious in the Yangtze River City Group, where the area converted from cultivated land to built-up land amounted to 4161.78 km^2^, accounting for 17.38% of the total cultivated land area in 1995. In the Jianghuai Ecological Economic Region, the water area increased with input from cultivated land, approximately 361.26 km^2^, accounting for 21.35% of the total area transferred out of cultivated land. Water area was the second largest LULC type to occupy the cultivated land, after built-up land. Water area also increased more obviously with transfers from cultivated land, grassland and built-up land in the Coastal Economic Belt. In addition, the transfers to cultivated land and built-up land resulted in an obvious decrease in grassland. Land transfer in the Huaihai Economic Region was less variable overall.

**Figure 4:**
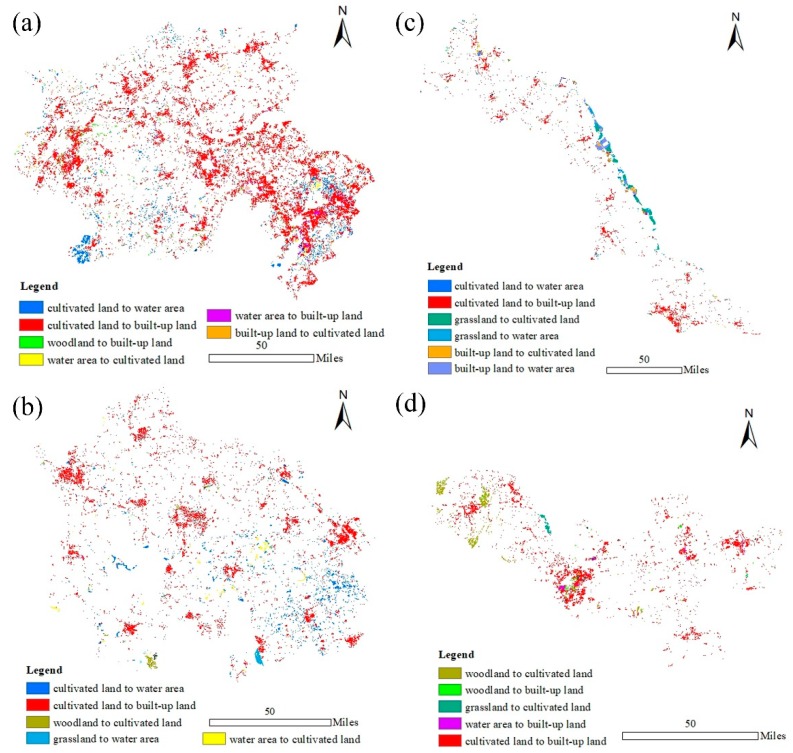
The spatial distributions of land use/land cover transfers from 1995 to 2015 in the four functional regions, where (**a**), (**b**), (**c**) and (**d**) represent the Yangtze River City Group, Coastal Economic Belt, Jianghuai Ecological Economic Region and Huaihai Economic Region, respectively.

Figure 4 shows the spatial distributions of the LULC transfers, the main land transfer types being chosen to show the distributions and the selected land transfer areas accounted for 93.47% of the total transferred area. The transfer of the cultivated land to built-up land presented regional differences. In the Yangtze River City Group, the transfer from cultivated land to built-up land was more obvious; it was distributed across the entire area and was concentrated in the southeast and west of the region in the form of large patches. However, in other functional regions, it was only densely distributed in certain areas in the form of small patches. Additionally, the cultivated land-water area presented a scattered distribution overall and was densely distributed in the southeast of the Jianghuai Ecological Economic Region. In the Coastal Economic Belt, the water area increased obviously. Land transfers from grassland and built-up land to the water area were mainly distributed in large patches along the coastline. Woodland-cultivated land was clustered in the form of large patches in the northwest of the Huaihai Economic Region. 

In summary, the LULC change trajectories of the four functional regions varied. As the economic engine of the whole province, the Yangtze River City Group presented the trajectory of other LULC types shifting to built-up land. The Coastal Economic Belt showed trajectories of cultivated land, grassland and built-up land shifting to water area and built-up land and grassland shifting to cultivated land along the coastline, reflecting the key regional development of the marine economy. The LULC changes in the Huaihai Economic Region are relatively flat, expanding the depth of the regional development. The Jianghuai Ecological Economic Region focuses on enhancing its ecological competitiveness with a trajectory of cultivated land and grassland shifting to water area.

### 3.4. Driving Forces of LULC Changes

Correlation analysis has been performed between land-use change and driving forces. When selecting drivers and assigning them to different land-use types, we follow rules to avoid repeatability. Table 6 shows that X_1_, and X_3_–X_8_ all negatively correlate with cultivated land area change (Y_1_) significantly. Only that the rural population correlates positively with cultivated land change (Y_1_). Transportation land (Y_2_) and residential and industrial land(Y_3_) both strongly correlated with these driving forces positively. The correlation between X_5_ and Y_2_ is stronger, with a correlation coefficient of 0.974, and the correlation coefficient between X_3_ and Y_2_ is relative lower, of 0.873. Y_3_ correlates with all driving forces strongly, with all correlation coefficients above 0.9.

### 3.5. Effects of LULC Changes on Carbon Storage in the Four Functional Regions

In total, LULC changes led to 714.03 × 10^4^ t of carbon storage loss for the whole province, and the four regions all presented decreasing carbon storage (Table 7). Among them, the Yangtze River City Group and the Huaihai Economic Region were the regions with the largest and least carbon storage losses, which were 387.93 × 10^4^ t and 57.04 × 10^4^ t, respectively. Carbon storage loss caused by the transfer of cultivated land to built-up land contributed the most to the total carbon storage loss, especially for the Yangtze River City Group, which contributed 84.75% of the total carbon storage loss. In the Coastal Economic Belt, the total carbon storage loss was 76.22 × 10^4^ t. The main LULC change trajectories of cultivated land, grassland and built-up land shifting to water area and built-up land and grassland shifting to cultivated land increased by 26.44 × 10^4^ t in total, which compensated the carbon storage loss to some extent. The carbon storage loss in the Jianghuai Ecological Economic Region was 192.84 × 10^4^ t and the main LULC change trajectories caused a total carbon loss of 39.48 × 10^4^ t.

## 4. Discussion

One of the main goals of this research was to attempt to find a way to promote low-carbon development among the four functional regions. Using the latest LULC raster data with a spatial resolution of 30 m, new soil survey data, and vegetation survey, we measured the soil and vegetation carbon densities of different LULC types in 2015, analyzed the diverse trajectories of the LULC changes and its driving forces, and compared the overall carbon storage changes between different trajectories over the period 1995–2015. Due to the inherent features of the four functional regions, the results presented obvious differences.

According to our study, the vegetation carbon density for woodland was the highest, which is due to woodland having the highest level of biomass [19,22]. Meanwhile, this can also increase the SOC level by returning more residuals into the soil [47,48]. For SOCD, we measured the SOCD using soil samples; this method has been widely used [43,49]. Water area presented the highest SOCD level, which was consistent with the published research [50]; this may be because silt in the water areas accumulate abundant organic matter through water transport mechanisms and because the decomposition rate underwater is low [51]. However, the vegetation carbon density for built-up land presented lower values, while its soil carbon densities were not low. This is because the built-up land surface was sealed and the sealed land cover can also prevent the release of soil carbon into the atmosphere for a short time. Thus, we believe more SOC research needs to be conducted. SOCD differs in different functional regions. Regarding inter-regional differences, the soil has abundant organic matter and its SOCD, therefore, presented a higher level in the Yangtze River City Group, whereas in the Coastal Economic Belt, sandy soil is widely distributed. The high sand content can greatly decrease the SOC accumulation [50] and the soil fertility is low, which can also harm the vegetation growth, lead to a lower biomass level compared with other regions, and can also reduce the vegetation residues returning into the soil. Thus, its SOCD presented lower values. 

LULC changes in the Jiangsu Province were obvious over the period 1995–2015, characterizing the built-up land expansion, especially obvious in the Yangtze River City Group and this was mainly caused by the socioeconomic development, which is consistent with previous studies [42,52]. Furthermore, this region is the economic engine of the whole province, possessing the majority of resources, such as high-quality education, medical treatment, and highly developed commerce and industry. Hence, these factors will attract more people to flow into this region and more built-up land is needed to feed residential living. According to previous studies, the land use policy may also strongly affect certain land use changes [53,54], which is also reflected in our study. For example, the land use changes presented densely along the coastline in the Coastal Economic Belt, which may be affected by the land use policy since the Jiangsu Province enacted a new coastal development plan in 2009 [49]. Additionally, the water area and cultivated land area increased obviously along the coastline due to the development of the aquaculture, as many land areas have been excavated to become fish ponds [55]. Focusing on developing ecological competitiveness in recent years, ecological parks developed rapidly in the Ecological Economic Region.

Overall, transfers of ecological land to built-up land could bring significant carbon loss. This is due to the large carbon density difference between built-up land and ecological land, and although the decrease of woodland is much lower than that of cultivated land, it also contributes to the obvious carbon storage loss because the high biomass in woodland vegetation always leads to the highest vegetation carbon density compared to other ecological lands [28,56,57]. Therefore, the protection of woodland is an effective way of increasing carbon storage. In addition, during the process of built-up land expansion, energy consumption will also increase, which will greatly increase the anthropogenic carbon emissions [58,59]. Regionally, the carbon storage loss in the Yangtze River City Group was the greatest because this region has the most obvious built-up land expansion, and, thus, the urban fringe area is the hotspot for future carbon storage losses and low carbon land management. Both technology and strategies support should especially be strengthened in such regions. The low carbon land management measures, such as intensive land use, land use structure optimization, green vegetation plantation, especially for high biomass trees, should be encouraged [38]. Carbon storage loss in the Huaihai Economic region was the lowest due to the lower built-up land expansion and the small SOCD difference between built-up land and cultivated land. In the Coastal Economic Belt, the transfer of grassland to cultivated land increased carbon storage, compensating for the regional carbon loss to some extent. Because most grass in this region is located within wetlands along the coastline, the transfer to cultivated land should be limited since wetlands have high ecological values, except for carbon accumulation, such as economy service value, cultural service, hydrological services, and climate improvement [60,61,62]. Our study also has some inevitable uncertainties. First, similar to most previous studies, this paper took 2015 as the base year for calculation and carbon densities were defined as a constant value: we did not consider the influence caused by vegetation growth. As for the vegetation carbon density of cultivated land, the carbon content varies with the carbon species, planting structure and climate change annually. Jiangsu Province has better hydrothermal conditions, fewer natural disasters, and crop production in this region are stable. We believe that this can reflect the carbon density of cultivated land. Second, soil sampling for water areas was mainly distributed in littoral shallow water areas, and deep water areas were not involved. Because LULC transfers mainly occurred in littoral areas, our calculation will not result in much bias for SOC changes caused by LULC changes. Third, because SOC in the surface soil layer is relatively high and the response to LULC changes is more sensitive [63], we only measured SOC in the 0–20 cm surface soil layer. If considering a 1 m deep soil layer, the SOC change caused by LULC changes may be greater. We did not consider the effects of vegetation carbon sinks during the growing season. We also did not consider the impact of SOC because a change in SOC takes longer than a change in the vegetation carbon storage [6], and a change in SOC is more complicated than a change in vegetation. Our calculations for SOC changes may have a certain error in comparison with the actual situation, and we, therefore, believe that SOC changes need further study. 

Overall, this study examined the influence of LULC changes on carbon storage and undertook a comparative analysis among the functional regions of Jiangsu Province. It enriches the LULC-induced carbon storage research from an inter-regional comparative perspective and may promote the coordinated development of the functional regions.

## 5. Conclusions 

### 5.1. Conclusions

LULC changes are of global concern because of their significance for the balance of carbon between terrestrial ecosystems and the atmosphere [64,65]. Comparative research into carbon storage changes induced by the LULC changes in functional regions of Jiangsu Province, one of the most developed provinces, helps in terms of understanding the development policy implications for the country’s other areas. Findings of this study were concluded as follows.

(1) LULC presented a more obvious change between 2005 and 2010. The built-up land expansion was obvious in four functional regions, especially in the Yangtze River City Group. Water area in both the Coastal Economic Belt and the Jianghuai Ecological Economic Region increased obviously. Woodland area in the Huaihai Economic Region decreased between 1995 and 2015, with the greatest rate of −22.62% during the period of 2005 to 2010.
(2) Between 1995 and 2015, approximately 10.26% of the entire province’s land area had its LULC types changed and expansion of built-up land and declining ecological land were the main LULC types change characteristics, which were mainly caused by socioeconomic development. Due to land use policy, the four functional regions also presented different LULC changes.
(3) The total carbon storage of the entire province decreased by 714.03 × 10^4^ t and the four regions all presented decreasing carbon storage. Cultivated land-built-up land is the main transfer type to carbon storage loss, with the decreased amount reaching 631.30 × 10^4^ t, accounting for 88.41% of the total carbon storage loss. The decrease in carbon storage was largest for the Yangtze River City Group (387.93 × 10^4^ t) because it presented the most obvious built-up land expansion. With much less built-up land expansion and a smaller SOCD difference between built-up land and cultivated land, the decrease in carbon storage in the Huaihai Economic Region was the least at 57.04 × 10^4^ t.

### 5.2. Policy Implications

By referring to both carbon storage changes induced by LULC changes and the existing inherent features of the four functional regions, this study suggests that low-carbon harmonious development overall needs to be determined by a master plan and policies for each functional region must be formulated according to the local conditions.

China’s 13th “Five-Year Plan” shows that maintaining green development is a fundamental national policy. In order to protect the environment and control carbon emissions, the ecosystem’s carbon storage can be increased as a whole by increasing the ecological land, controlling built-up land, optimizing land use structure, and reducing the disturbance of land use to balance the carbon storage. From the perspective of different functional regions, the Yangtze River City Group is the developed region of the province. In order to reduce carbon emissions, the Yangtze River City Group should adjust its energy structure, improve its energy development and utilization efficiency on the basis of controlling built-up land. The Coastal Economic Belt should pay attention to protecting ecological land and increasing the regional carbon storage, while vigorously developing its marine economy. The Jianghuai Ecological Economic Region has a natural ecological competitiveness. While building an ecological garden, it should adjust its land use structure, control water area, protect ecological land, and improve the ecological competitiveness of the province. Xuzhou should actively integrate development into all aspects and it should actively carry out industrial transformation, develop tertiary industries and strive to become the central city in the Huaihai Economic Region.

Defining the developmental orientation in each region is the primary requirement for lowering carbon emissions. The functional regions have their own positioning and mutual synergy, which is of great significance to the regional coordinated development and can improve the overall competitiveness of the Jiangsu Province.

## Figures and Tables

**Table 1:** The carbon storage and carbon density of each crop in the Jiangsu Province.

Category	Crop Species	H*_i_*	C*_f_*	Carbon Storage (Tg)	Carbon Density (kg/m^2^)
Food crops	Wheat	0.40	0.49	14.38	0.66
	Rice	0.45	0.41	17.79	0.78
	Corn	0.40	0.47	2.96	0.66
	Sorghum	0.35	0.45	0.00	0.96
	Millet	0.40	0.45	0.00	0.16
	Yam	0.70	0.42	0.20	0.37
	Soya bean	0.34	0.45	0.64	0.32
	Others	0.40	0.45	3.43	0.70
	Total	--	--	35.98	0.63
Cash crops	Cotton	0.10	0.45	0.53	0.56
	Peanut	0.43	0.45	0.37	0.41
	Rapeseed	0.25	0.45	1.91	0.51
	Sugar cane	0.50	0.45	0.04	2.76
	Tobacco leaf	0.60	0.50	0.00	0.14
	Others	0.42	0.45	0.02	0.20
	Total	--	--	2.85	0.50
Other crops	Vegetable	--	0.50	25.18	1.76
	Fruit	--	0.50	2.77	1.70
	Total	--	--	27.95	1.75

**Table 2:** The vegetation carbon densities of different land use/land cover (LULC) types in the Jiangsu Province.

LULC Type	Vegetation Carbon Density (kg/m^2^)	LULC Type	Vegetation Carbon Density (kg/m^2^)
Cultivated land	1.13	Water area	0.00
Woodland	1.94	Built-up land	0.11
Grassland	0.21	Unused land	0.00

**Table 3:** The soil organic carbon (SOC) densities of different LULC types in the four functional regions (kg/m^2^).

	Region	Yangtze River City Group	Coastal Economic Belt	Jianghuai Ecological Economic Region	Huaihai Economic Region
LULC Type					
Cultivated land		3.50	2.63	3.10	2.67
Woodland		3.57	2.44	2.82	2.87
Grassland		3.45	1.82	3.09	3.46
Water area		3.85	2.35	3.15	2.96
Built-up land		3.73	2.57	2.91	2.93
Unused land		3.46	1.80	2.96	3.41
Mean		3.59	2.27	3.00	3.05

**Table 4:** The amounts of LULC changes between typical years in the four functional regions.

Region	LULC Tape	Area (km^2^)				Change Rate (%)			
		1995–2000	2000–2005	2005–2010	2010–2015	1995–2000	2000–2005	2005–2010	2010–2015
Yangtze River City Group	Cultivated land	−488.19	−1035.46	−2580.30	−544.35	−2.04	−4.41	−11.51	−2.74
	Woodland	−31.01	−2.21	−50.96	−12.20	−1.64	−0.12	−2.75	−0.68
	Grassland	−3.98	−3.87	−19.14	20.80	−2.34	−2.34	−11.82	14.58
	Water area	53.99	225.04	224.51	−97.48	1.67	6.84	6.39	−2.61
	Built-up land	472.90	816.50	2358.55	632.46	10.52	16.44	40.78	7.77
	Unused land	−1.91	0.00	67.33	−1.04	−16.69	−0.02	706.86	−1.35
Coastal Economic Belt	Cultivated land	45.83	−48.94	−698.14	−2.78	0.24	−0.26	−3.70	−0.02
	Woodland	−1.27	−0.18	−27.48	−27.02	−0.38	−0.05	−8.15	−8.73
	Grassland	−141.27	−41.79	−101.33	−14.05	−29.03	−12.10	−33.39	−6.95
	Water area	62.93	3.43	220.66	−14.34	7.48	0.38	24.31	−1.27
	Built-up land	33.97	87.48	536.37	115.37	1.12	2.84	16.95	3.12
	Unused land	−0.14	0.00	69.91	−57.23	−100.00	0.00	0.00	−81.85
Jianghui Ecological Economic Region	Cultivated land	−159.68	−117.52	−1028.28	−223.82	−0.85	−0.63	−5.55	−1.28
	Woodland	−0.50	7.59	−48.64	−5.11	−0.12	1.75	−11.01	−1.30
	Grassland	−0.31	−1.36	−27.74	3.25	−0.19	−0.81	−16.60	2.33
	Water area	109.13	52.57	150.11	10.29	2.38	1.12	3.17	0.21
	Built-up land	52.59	58.72	945.13	213.69	1.28	1.41	22.43	4.14
	Unused land	−0.26	0.00	9.42	0.75	−100.00	0.00	0.00	7.91
Huaihai Economic Region	Cultivated land	−92.35	1.05	−250.26	−91.31	−1.23	0.01	−3.36	−1.27
	Woodland	−5.78	−0.01	−121.03	−2.15	−1.07	0.00	−22.62	−0.52
	Grassland	−0.63	0.00	−20.52	3.17	−1.30	0.00	−42.98	11.64
	Water area	6.07	−0.74	−14.94	0.99	1.21	−0.15	−2.94	0.20
	Built-up land	93.88	−0.30	405.49	86.87	4.92	−0.02	20.25	3.61
	Unused land	0.00	0.00	1.26	1.24	0.00	0.00	36.83	26.49

**Table 5:** The land transformation matrix for the Jiangsu Province between 1995 and 2015 (km^2^).

Region		2015	Cultivated Land	Woodland	Grassland	Water Area	Built-up Land	Unused Land	Total
	1995								
Yangtze River City Group	Cultivated land		19,174.32	43.74	16.20	602.64	4161.78	22.68	24,021.36
	Woodland		9.72	1732.59	0.00	4.05	104.49	30.78	1881.63
	Grassland		5.67	1.62	132.84	16.20	16.20	0.00	172.53
	Water area		58.32	0.81	15.39	2988.90	136.08	3.24	3202.74
	Built-up land		82.62	7.29	0.00	21.06	4365.09	5.67	4481.73
	Unused land		0.00	1.62	0.00	1.62	0.00	4.05	7.29
	Total		19,330.65	1787.67	164.43	3634.47	8783.64	66.42	33,767.28
Coastal Economic Belt	Cultivated land		17,814.33	8.10	9.72	92.34	937.98	3.24	18,865.71
	Woodland		45.36	278.64	0.00	0.00	16.20	1.62	341.82
	Grassland		195.21	0.00	154.71	106.92	34.02	0.00	490.86
	Water area		30.78	0.00	16.20	766.26	44.55	9.72	867.51
	Built-up land		73.71	0.00	8.10	172.53	2775.06	0.00	3029.40
	Unused land		0.00	0.00	0.00	0.00	0.00	0.00	0.00
	Total		18,159.39	286.74	188.73	1138.05	3807.81	14.58	23,595.30
Jianghui Ecological Economic Region	Cultivated land		17,113.68	4.86	1.62	361.26	1315.44	8.91	18,805.77
	Woodland		30.78	382.32	0.00	4.86	11.34	0.00	429.30
	Grassland		0.81	0.00	144.18	30.78	0.81	0.00	176.58
	Water area		52.65	0.81	0.00	4503.60	21.06	4.05	4582.17
	Built-up land		46.17	0.00	0.81	7.29	4035.42	0.81	4090.50
	Unused land		0.00	0.00	0.00	0.00	0.00	0.00	0.00
	Total		17,244.09	387.99	146.61	4907.79	5384.07	13.77	28,084.32
Huaihai Economic Region	Cultivated land		6980.58	2.43	1.62	13.77	553.23	1.62	7553.25
	Woodland		103.68	388.80	0.00	0.00	29.16	0.00	521.64
	Grassland		14.58	0.00	31.59	0.00	6.48	0.00	52.65
	Water area		4.86	0.00	0.00	478.71	19.44	1.62	504.63
	Built-up land		12.15	1.62	0.00	3.24	1895.40	0.81	1913.22
	Unused land		0.00	0.00	0.00	0.00	0.00	3.24	3.24
	Total		7115.85	392.85	33.21	495.72	2503.71	7.29	10,548.63

**Table 6:** The correlation coefficients between land-use changes and driving forces.

	Y_1_	Y_2_	Y_3_		Y_1_	Y_2_	Y_3_
**X_1_**	–0.903 **	0.957 **	0.984 **	**X_6_**	–0.955 **	--	0.967 **
**X_2_**	--	0.884 **	0.954 **	**X_7_**	–0.941 **	--	0.927 **
**X_3_**	–0.942 **	0.873 **	0.917 **	**X_8_**	0.938 **	--	--
**X_4_**	–0.949 **	--	--	**X_9_**	--	0.934 **	--
**X_5_**	–0.895 **	0.974 **	0.986 **	**X_10_**	--	0.917 **	--

Significant at ^∗∗^ *p* < 0.01 levels.

**Table 7:** The carbon storage transfer matrix for the Jiangsu Province between 1995 and 2015 (×10^4^ t).

Region		2015	Cultivated Land	Woodland	Grassland	Water Area	Built-up Land	Unused Land	Total
	1995								
Yangtze River City Group	Cultivated land		0.00	3.85	−1.57	−47.01	−328.78	−2.65	−376.16
	Woodland		−0.86	0.00	0.00	−0.67	−17.45	−6.31	−25.29
	Grassland		0.55	0.30	0.00	0.31	0.29	0.00	1.45
	Water area		4.55	0.13	−0.29	0.00	−0.14	−0.13	4.13
	Built-up land		6.53	1.22	0.00	0.02	0.00	−0.22	7.55
	Unused land		0.00	0.33	0.00	0.06	0.00	0.00	0.40
	Total		10.77	5.83	−1.86	−47.29	−346.07	−9.31	−387.93
Coastal Economic Belt	Cultivated land		0.00	0.50	−1.68	−13.02	−101.30	−0.64	−116.14
	Woodland		−2.81	0.00	0.00	0.00	−2.75	−0.42	−5.98
	Grassland		33.77	0.00	0.00	3.42	2.21	0.00	39.40
	Water area		4.34	0.00	−0.52	0.00	1.47	−0.53	4.76
	Built-up land		7.96	0.00	−0.53	−5.69	0.00	0.00	1.74
	Unused land		0.00	0.00	0.00	0.00	0.00	0.00	0.00
	Total		43.26	0.50	−2.73	−15.29	−100.37	−1.59	−76.22
Jianghui Ecological Economic Region	Cultivated land		0.00	0.26	−0.15	−39.02	−159.17	−1.13	−199.21
	Woodland		−1.63	0.00	0.00	−0.78	−1.97	0.00	−4.39
	Grassland		0.08	0.00	0.00	−0.46	−0.02	0.00	−0.41
	Water area		5.69	0.13	0.00	0.00	−0.27	−0.08	5.47
	Built-up land		5.59	0.00	0.02	0.09	0.00	0.00	5.70
	Unused land		0.00	0.00	0.00	0.00	0.00	0.00	0.00
	Total		9.72	0.39	−0.13	−40.17	−161.44	−1.21	−192.84
Huaihai Economic Region	Cultivated land		0.00	0.25	−0.02	−1.16	−42.05	−0.06	−43.04
	Woodland		−10.47	0.00	0.00	0.00	−5.16	0.00	−15.63
	Grassland		0.19	0.00	0.00	0.00	−0.41	0.00	−0.22
	Water area		0.41	0.00	0.00	0.00	0.16	0.07	0.64
	Built-up land		0.92	0.29	0.00	−0.03	0.00	0.03	1.21
	Unused land		0.00	0.00	0.00	0.00	0.00	0.00	0.00
	Total		−8.95	0.53	−0.02	−1.18	−47.46	0.04	−57.04
